# Electron Tomography of the Contact between T Cells and SIV/HIV-1: Implications for Viral Entry

**DOI:** 10.1371/journal.ppat.0030063

**Published:** 2007-05-04

**Authors:** Rachid Sougrat, Alberto Bartesaghi, Jeffrey D Lifson, Adam E Bennett, Julian W Bess, Daniel J Zabransky, Sriram Subramaniam

**Affiliations:** 1 Laboratory of Cell Biology, National Cancer Institute, National Institutes of Health, Bethesda, Maryland, United States of America; 2 AIDS Vaccine Program, SAIC-Frederick, National Cancer Institue, Frederick, Maryland, United States of America; King's College London, United Kingdom

## Abstract

The envelope glycoproteins of primate lentiviruses, including human and simian immunodeficiency viruses (HIV and SIV), are heterodimers of a transmembrane glycoprotein (usually gp41), and a surface glycoprotein (gp120), which binds CD4 on target cells to initiate viral entry. We have used electron tomography to determine the three-dimensional architectures of purified SIV virions in isolation and in contact with CD4+ target cells. The trimeric viral envelope glycoprotein surface spikes are heterogeneous in appearance and typically ∼120 Å long and ∼120 Å wide at the distal end. Docking of SIV or HIV-1 on the T cell surface occurs via a neck-shaped contact region that is ∼400 Å wide and consistently consists of a closely spaced cluster of five to seven rod-shaped features, each ∼100 Å long and ∼100 Å wide. This distinctive structure is not observed when viruses are incubated with T lymphocytes in the presence of anti-CD4 antibodies, the CCR5 antagonist TAK779, or the peptide entry inhibitor SIVmac251 C34. For virions bound to cells, few trimers were observed away from this cluster at the virion–cell interface, even in cases where virus preparations showing as many as 70 envelope glycoprotein trimers per virus particle were used. This contact zone, which we term the “entry claw”, provides a spatial context to understand the molecular mechanisms of viral entry. Determination of the molecular composition and structure of the entry claw may facilitate the identification of improved drugs for the inhibition of HIV-1 entry.

## Author Summary

Retroviruses such as simian immunodeficiency virus and HIV-1 enter target cells by exploiting the interaction between their surface glycoproteins and cell surface receptors. Knowledge of the structures of these glycoproteins and of the molecular details of their interaction with cell surface receptors is of fundamental interest in understanding viral entry mechanisms. Electron tomo-graphy is a powerful approach to determining the three-dimensional structures of large and heterogeneous sub-cellular assemblies such as virus–cell contact regions that cannot easily be analyzed by high-resolution structural methods such as X-ray crystallography. Here, we have used electron tomographic approaches to show that SIV and HIV-1 viruses make contact with T cells via a unique structure that we term the viral “entry claw”, which is typically composed of about six clustered rods of density that span the contact region. Investigation of the structure of the entry claw and the factors that promote its formation could lead to new insights into the design of more effective drugs to inhibit HIV entry.

## Introduction

Entry of HIV-1 into target cells involves the interaction of the surface glycoprotein gp120 (designated SU) with the cell surface receptor CD4 [1], a binding-induced structural change [2] in gp120 that creates the binding site for a cellular seven-transmembrane-helix co-receptor protein [3], followed by conformational changes [4] in the transmembrane glycoprotein gp41 (designated TM) that allow formation of the “pre-hairpin” conformation [5,6]. Insertion of the fusogenic portion of the TM polypeptide into the target cell membrane ultimately leads to fusion of the viral and target cell membranes [7]. Substantial insights into the entry mechanism have come from cell and structural biological studies, which suggest that the molecular species that initiates the steps leading to fusion is a trimer of envelope glycoprotein heterodimers [8].

Electron tomography is a powerful approach for determining the three-dimensional (3-D) structures of large and heterogeneous sub-cellular assemblies at resolutions that are typically one to two orders of magnitude higher than those that can be currently achieved using light microscopy [9–11]. Because these assemblies are not generally amenable to analysis by crystallographic approaches, electron tomography provides tools to bridge the gap between cellular and molecular structure. Although the highest resolutions have been obtained from tomographic analyses of thin, unstained specimens in a near-native state at cryogenic temperatures, significant information has also been derived from tomographic imaging of chemically fixed and stained specimens that allows structural investigation of the interior of thick cellular specimens. Here, we have used electron tomographic approaches to analyze the 3-D architectures of simian immunodeficiency virus (SIV) and HIV-1 virions incubated with CD4+ T lymphocyte target cells to identify structural features of cell-bound viruses trapped at a stage prior to entry into target cells.

## Results

**Figure 1:**
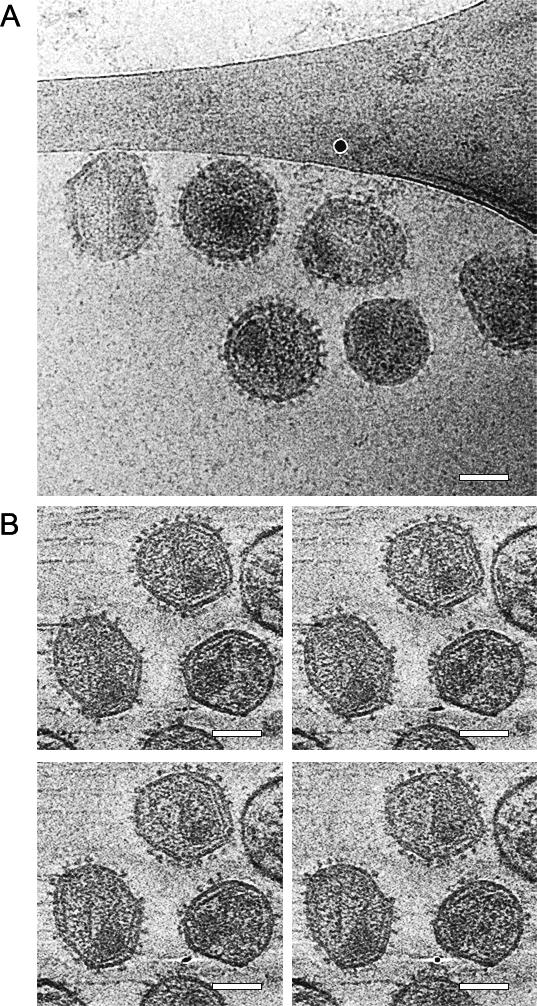
Analysis of Purified SIV Virions by Cryo-Electron Tomography (A) Low-dose projection image of a plunge-frozen specimen. (B) A series of images recorded over a tilt range of −63° to +63° was used to reconstruct a 3-D volume of vitrified viruses similar to those shown in (A). Four 1-nm-thick tomographic slices at different depths from a tomogram of plunge-frozen SIV virions are shown. Scale bars are 50 nm long. The dark black spots are from 5-nm-wide gold fiducial markers used for aligning individual images in a tilt series.

To validate the approach used for structural analysis of infected cells using electron tomography of stained, plastic-embedded specimens, we first carried out analysis of unstained purified viruses using cryo-electron tomography, and compared the resulting 3-D structures with those obtained from analysis of free virions in stained, plastic-embedded specimens. For ease of comparative analysis, we used an SIV strain that expresses a high level of envelope glycoprotein as a consequence of the effects of the 9-kDa C-terminal truncation in TM [12,13]. Purified virion samples were rapidly vitrified by plunge-freezing in liquid ethane cooled to ∼ −180 °C. The frozen hydrated specimens were imaged at liquid nitrogen temperatures using a 300 kV electron microscope equipped with a field emission gun and an energy filter. Four 1-nm-thick slices from a reconstructed tomogram are shown in Figure 1. The images reveal the distribution of the envelope glycoprotein spikes on the surface and a glimpse of the internal core of the virus. Since these are from unstained virus preparations, the contrast in the images arises from the intrinsic distribution of mass in the virus.

**Figure 2:**
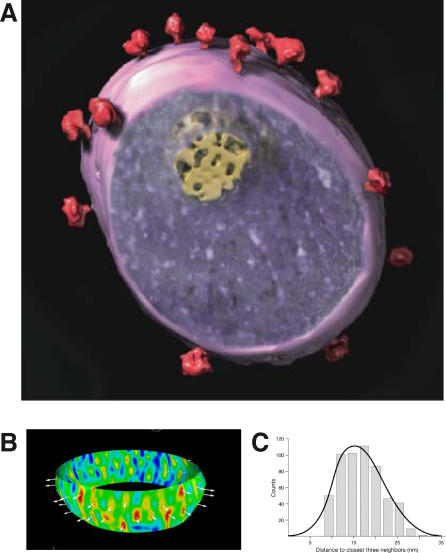
Analysis of 3-D Structure of SIV (A) Segmented representation of the surface and interior of a single virus from the data shown in Figure 1. For the sake of clarity, only selected regions of the interior and exterior are highlighted: elements shown include the viral envelope (magenta), viral spikes (red), core (yellow), and interior (translucent purple). (B) Surface plot showing automated detection of spikes on the surface of the viral membrane. (C) Nearest neighbor analysis to measure the distribution of distances between neighboring spikes.

A segmented 3-D rendering of one of the four virions shown in Figure 1 is presented in Figure 2A, with the surface spikes and the internal core highlighted. Substantial variation was evident between virions in the numbers of glycoprotein spikes on the surface, with some displaying as many as 100 spikes, while others were largely devoid of spikes. Inspection of the 3-D structures of several individual viral spikes shows that they have shapes that are often narrow (∼60 Å) in the region closest to the membrane, and wider (∼120 Å) at the distal end. This size is consistent with that expected from a trimer of envelope glycoprotein molecules protruding from the surface of the viral membrane, with the TM trimer packed in the narrower end of the viral spike. There is noticeable structural heterogeneity in individual spikes, which could arise either from the low signal-to-noise ratios inherent to cryo-electron tomography, the effect of the missing wedge in data collection, and/or from genuine conformational variability in the viral spikes [14]. To evaluate the distribution of the viral spikes on the surface of the virus, we developed automated feature extraction tools to identify the locations of individual spikes, as illustrated in Figure 2B. In this SIV preparation, spikes are distributed throughout the surface without obvious clustering, with an average spacing of ∼200 Å between neighboring spikes (Figure 2B).

Images of individual spikes can be averaged together with compensation for the missing wedge to improve the signal-to-noise ratio; however, an averaged structure does not capture the structural heterogeneity in spike structure, and the resulting averaged image can vary depending on the approach used to align the spikes to each other. Different averaged structures and differing atomic models for the SIV spike have been recently reported by Zhu et al. [15] and by Zanetti et al. [16] (see commentary in [14]). Here, we show the structures of SIV envelope glycoprotein spikes without averaging solely to provide a reference for the subsequent structural analysis of viruses captured in contact with target cells. The viruses we (Figure 1) and Zhu et al. [15] have imaged are variants of SIVmac239 with a truncated TM glycoprotein. The SIVmneE11S virus analyzed by Zanetti et al. [16] is closely related; however both sets of viruses have high levels of envelope glycoproteins as a consequence of truncation of the cytoplasmic tail of the TM glycoprotein.

**Figure 3:**
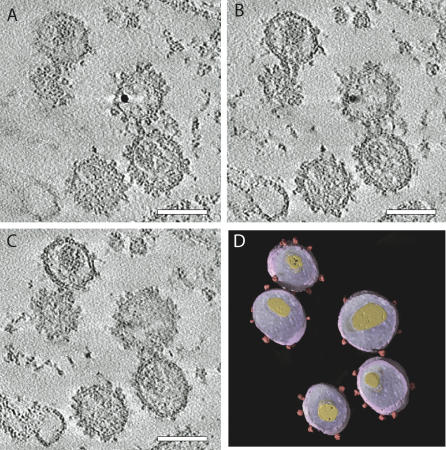
Structural Analysis of Virions from an SIV-Infected T Cell Preparation (A–C) Three 1-nm slices at different depths from a tomogram of a 150-nm-thick section of fixed, plastic-embedded T cell shows virions in the extracellular space. (D) 3-D surface rendering of the virions shown in (A–C). The size and appearance of these viruses imaged in fixed, plastic-embedded cells at room temperature is closely comparable to those observed in vitrified specimens of purified viruses analyzed by low-dose electron tomography at liquid nitrogen temperatures. Scale bars are 100 nm long in all panels.

To compare the overall architecture of viral spikes observed by cryo-electron tomography of purified SIV virions to that seen in fixed, plastic-embedded SIV, we obtained tomograms from 150-nm-thick sections prepared from plastic-embedded specimens of SIV-infected T cells. The outlines of viruses can be readily identified in single slices from these cellular tomograms (Figure 3A–3C) and in the segmented representation of the tomogram (Figure 3D). The spikes on the surface of these virions are ∼120 Å high and similar in appearance to those obtained from unstained viruses obtained using low-dose cryo-electron tomography. While the tomograms from stained specimens report on the 3-D distribution of the stain rather than the intrinsic density of the virus, the demonstration that viral spikes analyzed by both methods have similar dimensions provides confidence for interpretation of tomograms of contact regions between virions and the surface membrane of target cells obtained using fixed, plastic-embedded specimens, as described below.

**Figure 4:**
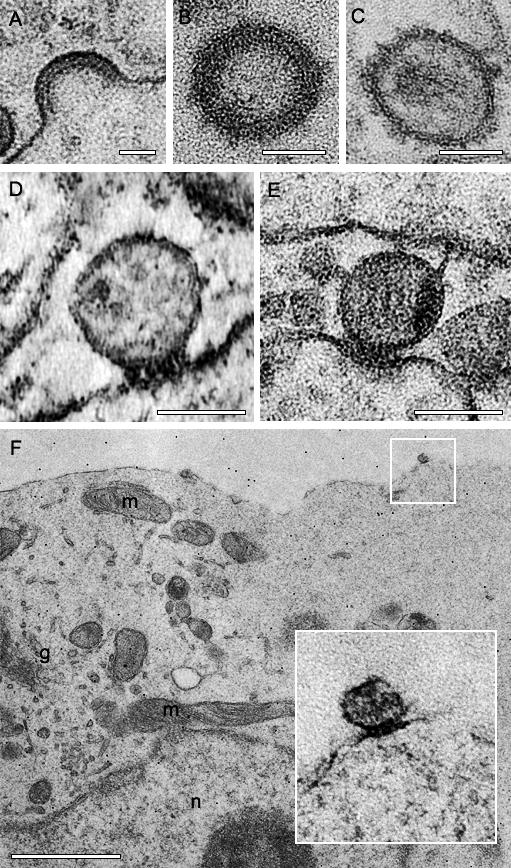
Projection Images from SIV-Infected Cells and from T Cells Exposed to SIV for Short Periods (A–C) Electron microscopic images of budding (A), immature (B), and mature (C) SIV particles in chronically infected cell suspensions. Scale bars are 50 nm long. (D and E) Distinct virus–cell contacts in chronically infected cells that are defined by a characteristic density at the interface between the virus and the cell membrane, and distinct from virus morphologies seen in (A–C). Note that in some instances (E), the curvature of the cell membrane follows the curvature of the virus where the contact is made. Scale bars are 100 nm long. (F) Projection image (higher magnifications shown in inset) of the contact region between SIV virions and T lymphocytes fixed after incubation at 37 °C for 15 min. At lower magnifications the overall context of the cell in the vicinity of the contact region is shown, while at the higher magnifications, entry claw contacts can be recognized in the projection view. Scale bar is 1 μm long. G, part of the Golgi ribbon; m, mitochondria; n, nucleus.

In addition to seeing mature virus particles with characteristic morphologies [17] in the extracellular medium (Figure 3), we also detected, at a lower frequency, regions of the cell surface where viruses appeared to be captured in close contact with the cell membrane (Figure 4D). This contact region, as visualized in projection, displayed a characteristic pattern of striated densities connecting the viral membrane to the cell membrane. In some instances, the (concave) curvature of the cell membrane was found to follow the (convex) curvature of the viral membrane at the region of contact (Figure 4E). The viruses involved in these contacts generally showed evidence of mature cores and did not display the characteristic thick layer of uncleaved Gag protein seen in immature viruses (Figure 4B), indicating that they were not immature virions in the process of budding (Figure 4A) or free mature viruses (Figure 4C).

To evaluate virus–cell contact regions potentially involved in infection events more unambiguously, we pre-incubated uninfected CD4+ T cells susceptible to SIV and HIV-1 infection with high concentrations of infectious virus at 4 °C, then warmed the cells to 37 °C and carried out rapid fixation after waiting for periods ranging from 15 min to 3 h. The rationale for pre-incubation at 4 °C was to allow binding, but not fusion, of virions to target cells, while warming to 37 °C was intended to allow progression of fusion and virus entry over time scales not long enough for viral replication [18]. Images of the virus–cell interface from these acutely infected cells (Figure 4F) display exactly the same type of cell–surface contact observed in the chronically infected cells (Figure 4D and 4E); the profiles shown are representative of the images obtained from over 200 different virus–cell contact regions imaged in cells fixed at different times after warming. We conclude from these observations that the contact zones found in chronically infected cells (Figure 4D and 4E) are therefore likely to represent infection events involving mature viruses binding back to target cells.

**Figure 5:**
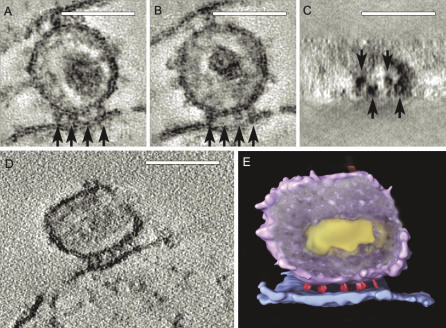
Electron Tomography of Virus–Cell Contact in Chronically and Acutely Infected Cells (A–C) Single 1-nm slices extracted from a dual axis tomogram reconstructed by weighted back-projection where part of an entry claw is captured in a chronically infected cell. One combination of three rods can be seen in one plane (A), while a different combination of three rods can be seen in the other (B). Four of the rods can be seen clearly in orthogonal view sectioned close to the plane of contact between virus and cell (C), with the densities arranged in a zig-zag manner. The black arrows point to the four densities in both transverse and top views. (D) A 1-nm tomographic slice from SIV–T cell contact region in cells fixed 15 min after warming to 37 °C following incubation with high viral concentrations. Scale bars are 100 nm long in (A–D). (E) 3-D rendering of the same contact region presented in panel (D), showing the viral envelope (magenta), contact rods (red), core (yellow), and cell membrane (blue). Note that there are almost no spikes on the virion surface away from the region of viral–cell contact.

Next, we investigated the 3-D spatial architecture of a typical virus–cell contact region such as those shown in Figure 4D and 4E using electron tomography. Two transverse views of a 3-D reconstruction of an entry claw in a chronically infected cell (at different depths in the tomogram) are shown in Figure 5A and 5B, while a top view along the plane of contact is presented in Figure 5C. Distinct rods of density in the contact region spaced at ∼150 Å can be observed, as indicated by the arrows. The same type of contact structure is also observed in cells subjected to acute infection, as shown in the tomographic slice (Figure 5D) and segmented version of the entire 3-D volume (Figure 5E) of a virus–cell contact in acute infection. Based on our findings, and its probable connection to viral entry (discussed below), we refer to this contact region as the viral “entry claw”. Between five and seven rods of density, arranged in a closely packed pattern, were typically observed in the entry claws that we imaged. Of the fourteen tomograms of entry claws that we analyzed in detail in 3-D, one was observed with four rods of density, seven were comprised of five rods, five had six rods, and two had seven rods. These findings are supported by visual inspection of these contact regions recorded in 2-D projection views. The variation in claw profile could arise, at least in part, due to the fact that not all contact regions were fully captured in the 100-nm-thick sections. Overall, each rod is ∼100 Å long and ∼100 Å wide with a center-to-center distance ranging from 140 Å to 170 Å between individual rods. The overall width of the claw ranges from ∼350 Å to 450 Å and forms the outlines of a neck-shaped region at the junction of the viral and target cell membranes. The spacing of these rods is slightly closer than the average spacing of envelope glycoprotein spikes observed in free viruses (∼200 Å), suggesting that rearrangement of spikes within the viral membrane may be involved in formation of the structure.

A unique feature observed in association with most viral entry claws we imaged was the virtual absence of visible spikes on the rest of the viral surface. This is remarkable given that the SIV viruses used in some of our studies display as many as ∼100 spikes distributed over their surface upon release from infected cells (Figures 1 and 2). We believe that a likely explanation of our observations is that the spikes remote from the region of contact may have been shed from the particles. It is formally possible, although improbable, that there exists a small percentage of viruses that only have a few viral spikes, and these are the ones that are preferentially engaged in entry claw formation. We cannot resolve at present whether both gp120 and the TM glycoprotein have been lost from virions involved in these docking interactions, or whether only gp120 has been lost, leaving the TM glycoprotein behind.

**Figure 6:**
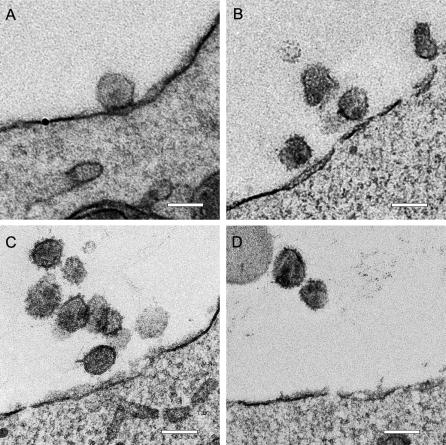
Analysis of Virus–Cell Contact under Different Conditions (A) Projection image of entry claw formed between SIV mac239 wild-type (full-length tail) and T cells fixed after incubation at 37 °C for 3 h. (B) Projection image recorded from samples where SIV mac239 tail-truncated virus was fixed without warming to 37 °C. While viruses could be occasionally found in close proximity of the cell membrane, no entry claw–like structures were observed. (C and D) Projection images recorded from cells incubated with SIV mac239 tail-truncated virus in the presence of 10 μM TAK779 or 5 μM C34 peptide, respectively. In both cases the viruses were incubated with cells for 3 h at 37 °C, and no entry claw–like structures could be detected. Scale bars are 100 nm in all panels.

We carried out a number of additional control experiments to verify that the virus–cell contact shown in Figures 4 and 5 represent specific contact events relevant to viral entry. The typical frequency of entry claw observation in our experiments was ∼ten events/300 imaged cell sections (corresponding to ∼15 entry claws/cell, assuming an ∼100-nm section and an average cell radius of ∼10 μm). When incubation of cells with virus was carried out in the presence of an anti-CD4 antibody known to block viral entry (anti-Leu3a; [19]), no instance of viral contact was found despite extensive screening of several hundred cells, indicating that the presence of the antibody inhibited close interactions of the kind seen in Figure 4. We conclude that cell surface CD4 is required for formation of the unique architecture observed in the contact region. Entry claw structures similar to those observed in Figures 4 and 5 were observed when we used a wild-type SIV mac239 virus expressing full length TM glycoprotein (Figure 6A). The average number of spikes in this viral isolate is known to be about ten times less than those in the tail-truncated version [13]. When cells were fixed only after the 4 °C incubation, and without warming to 37 °C, no evidence of entry claw formation was found (0/300 imaged cells), even in instances where viruses were observed in proximity of the cell membrane (Figure 6B). While viral binding to the cell surface is not expected to be restricted to the period of incubation at 4 °C, the lack of entry claw formation in the absence of warming suggests that the latter step is required for its stabilization. Similarly, no entry claw structures were found when incubation of viruses was carried out in the presence of the CCR5 antagonist TAK779 (Figure 6C) or the peptide inhibitor C34 (Figure 6D). Together, these experiments provide strong evidence that formation of the entry claw requires interaction of the envelope glycoprotein and the cell surface receptors known to be involved in viral entry.

**Figure 7:**
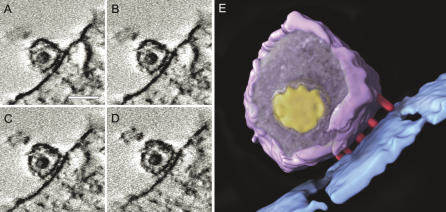
Imaging of HIV-1 in Contact with T Cells (A–D) Four slices at different depths in a tomogram of the contact between HIV-1 and T cells, with cells fixed following incubation for 1 h at 37 °C. Scale bar is 100 nm long. (E) 3-D surface rendering of the tomographically derived architecture of the contact region between HIV-1 and the T cell membrane shown in panels (A–D), with color scheme as in panel (E) of Figure 5.

To determine whether the structural features observed in the contact region are unique to the SIV used in our studies, we analyzed the virus–target cell contact region after incubating CD4+ target cells with HIV-1_MN_ virions. Cells were processed using the same approaches employed for the SIV studies described above. 2-D and 3-D images from the HIV-1-infected cells (Figure 7) demonstrate that the same type of architecture that is observed for contact between SIV and T cells is also observed for contact of HIV-1.

**Figure 8:**
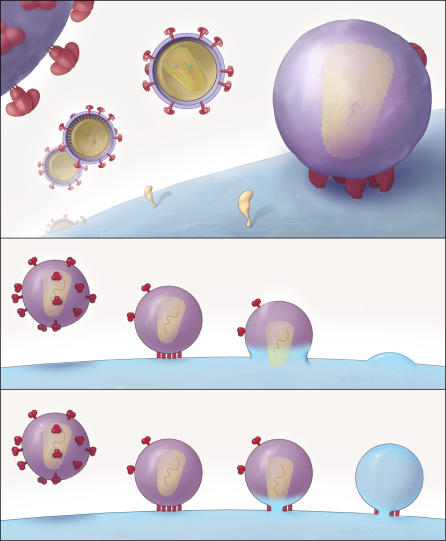
Schematic Representation of the Key Structural Features of SIV and HIV-1 Entry into T Cells (A) Different stages of viral entry from budding, to maturation, to entry claw formation. For the SIV strain used here, viruses that are docked to the cell via an entry claw show very few, if any, viral spikes on their surface, whereas non-contacting viruses typically display between 60 and 100 spikes on their surface. The entry claw is composed of between five to seven anchors spanning the region between the virus and the cell, each ∼100 Å long, and spaced laterally by ∼150 Å. (B and C) Two alternative models for viral entry. In the global fusion model (B), the formation of the entry claw is followed by progressive fusion of the viral membrane across its width, leading to merger of the contents of the viral membrane with the cellular membrane. In the local fusion model (C), the formation of the entry claw is followed by the creation of a local pore centered at one of the rods, leading to delivery of the viral core into the cell.

Figure 8A provides a schematic interpretation of the tomographic analysis presented here, showing viruses in different stages of maturation and in contact with T cells to form the entry claw. Density corresponding to the core was observed in some but not all viruses engaged in entry claw formation after both short and long periods of incubation, although we note that since many contacting viruses would only have been captured partially in the 100-nm sections, the absence of core-like density cannot be used as definitive diagnosis for the absence of a core in the contacting viruses. Our working hypothesis is that each of the rods is derived from a single viral spike, although their precise molecular composition remains to be determined. The spacing of these rods is slightly closer than the average distribution of envelope glycoproteins observed in free viruses, suggesting that rearrangement of spikes within the viral membrane could be involved. Although extensive labeling experiments will be necessary to determine the precise composition of the entry claw, it is interesting to note that that the observed average length (∼100 Å) of the rods of density connecting the viral and cell membranes is consistent with the expected dimensions of a potentially fully extended state of TM, representing the pre-hairpin intermediate [6].

## Discussion

Viral binding and entry are key processes in the biology of AIDS virus infection and represent critical steps for potential prophylactic intervention by vaccine-induced neutralizing antibodies, or therapeutic intervention by inhibitors of binding or viral fusion. The observations we present here of the size and composition of the SIV and HIV-1 entry claw are in agreement with previous studies suggesting that viral entry is likely to be mediated by an oligomeric assembly of viral spikes and cell surface receptors. Experimental support for the cooperative interaction of multiple envelope glycoproteins in the entry of influenza [20], Semliki Forest virus [21], rabies virus [22], and baculovirus [23] has already been documented. In the case of HIV, there is also evidence that between four and six co-receptors (CCR5/CXCR4) and multiple CD4 molecules are likely to be present at the fusion pore [24,25]. The combination of these studies and the results of our structural analysis suggest that the entry claw is a specific macromolecular structure associated with initiating fusion and viral entry.

What stage of the fusion does the entry claw represent? Some models for viral fusion generally invoke the formation of a wide-necked pore via a hemi-fusion intermediate [26]. Despite thorough screening, we have not yet detected structures that appear to represent this type of fusion event where one might expect a partially fused virion in the act of transferring the viral genome-containing core into the cell. One possible explanation for this could be that the fusion event itself is very rapid, and therefore not detected in our experiments. In support of this idea, there is considerable evidence that once viruses bind to target cells, formation of sufficient numbers of ternary complexes of Env, CD4, and co-receptor is likely to be the rate-limiting step in viral entry [27]. This initial stage of viral–cell interaction corresponds at the biochemical level to generation of the pre-hairpin intermediate [6], and at the cellular level to the “temperature-arrested stage” [28], where viruses can remain attached to cells at temperatures that are suboptimal for fusion. In this model (Figure 7B), steps subsequent to initial docking must include, at a minimum (although not necessarily in this sequence), helix bundle formation, creation of the correct geometry for fusion, formation of a hemi-fusion intermediate leading to merger of the outer leaflets of the viral and target cell membranes, and the fusion event itself, which results in delivery of the viral genome into the cell.

There is an alternative model (Figure 7C) for how fusion might occur that is potentially consistent with recent studies that have concluded that a single fusion competent envelope glycoprotein trimer may be sufficient to support HIV-1 entry [29]. At first glance, these studies would appear to contradict the findings reported here, which consistently show multiple viral spikes acting in concert to form the entry claw. However, the key point is that in the analysis carried out by Sodroski and co-workers [29], the number of functional, fusion-competent trimers was modulated by varying the relative proportion of functional and defective (fusion-incompetent) glycoproteins present on surface of the virus, without alteration in the average number of spikes per virion. These non-functional envelope glycoproteins were mutants fully capable of CD4 binding, but defective for cleavage to generate fusogenic TM polypeptides. Thus, multiple trimers might participate in formation of an entry claw structure, creating a stabilized scaffold to dock the virus to the cell membrane, regardless of their fusion competence. If fusion were to occur via local puncture of the membrane at the location of the single functional spike, presumably via the formation of a fusogenic, six–helix bundle intermediate [6], it could account both for the structural role of multiple viral spikes in forming the claw to create the scaffold to dock the virus to the cell membrane, and the finding that one functional trimer is adequate for viral entry. The electron tomographic analyses show that dimensions of the pore that could be formed between the anchors of the claw could be as wide as ∼300 Å. An opening of this size is comparable to the dimensions of an intact viral core [30] and may thus be large enough to transport the core of the virus into the cell even without invoking any additional pore expansion. In this mechanism, the entry claw scaffold and the viral envelope could continue to remain attached on the cell surface after loss of viral core. Although evidence of staining from viral cores was observed in entry claw contacts after both short and long incubation periods, at present, we cannot definitively distinguish between the two models in Figure 8.

The discovery of the entry claw raises many fundamental questions about viral entry. What is the molecular composition and stoichiometry of the components forming the entry claw? Do other complex factors such as membrane reorganization and actin rearrangement play a role in its formation or stabilization? Is persistence of the entry claw dictated by the speed of the topological rearrangements needed for formation of the helix bundle that triggers membrane fusion? Does the composition of the claw change following initial contact? Are there other structural intermediates that can be trapped by using selected gp120 or gp41 mutants with altered functional properties, or by the addition of antibodies or ligands to the cell surface receptors involved? Can the fusion event itself be captured and visualized? What is the role, if any, of interactions between the C-terminal tail of the envelope glycoprotein (TM) and the Gag matrix in rearrangements of viral spikes at the point of contact? Continuing improvements in technologies for cryo-electron tomography, combined with X-ray crystallographic analysis of the envelope glycoprotein in its various conformations, offer the promise of making significant advances towards answering these questions.

## Materials and Methods

### 3-D imaging of purified viruses.

Viruses were isolated from chronically infected cells by density gradient centrifugation and further purified by using an anti-CD45 affinity column to remove contaminating microvesicles [31]. Purified viral suspensions were deposited on Quantifoil grids (Quantifoil, http://www.quantifoil.com), mixed with a solution of colloidal gold, and plunge-frozen using a Vitrobot device (FEI Company, http://www.vitrobot.com). Grids were imaged at liquid nitrogen temperatures using a Polara field emission gun electron microscope (FEI Company) equipped with a 2k × 2k CCD placed at the end of Gatan energy filter, and operated at 300 kV. Tilt series for tomographic reconstruction were acquired using the FEI tomography software package and reconstructed using either weighted back-projection or SIRT procedures as implemented in IMOD [32] and Inspect 3D (FEI Company), respectively. Typically, for the cryo-electron tomographic experiments, electron doses of ∼120 electrons/Å^2^ were used, and images were collected at 2.5-degree intervals. Visualization and semi-automated segmentation was carried out using software tools implemented in the program Amira (TGS, http://www.tgs.com).

### 3-D imaging of viruses in chronically and acutely infected cells.

For acute infection studies using SUPT1/CCR5 CL.30 cells as targets, replicate aliquots of cells were resuspended at 5 × 10^5^ cells in 200 μl in microcentrifuge tubes in RPMI 1640 with 10% (v/v) heat-inactivated fetal calf serum. The cells of this human T cell line naturally express CD4 and CXCR4 and have been engineered by retroviral transduction to express CCR5 [33]. Cells were incubated at 4 °C for 1 h, with or without monoclonal anti-Leu3a (Becton Dickinson, http://www.bd.com) antibody at a final concentration of 10 μg/mL. Pre-cooled, sucrose gradient purified concentrated SIV or HIV-1 was then added to the cells, using approximately 2 μg of p28^CA^ or 1 μg of p24^CA^ equivalent per 5 × 10^5^ cells (corresponding to approximately 1 × 10^10^ to 2 × 10^10^ virions and a nominal multiplicity of infection of between 0.1 and 1, based on titration in SUPT1/CCR5 CL.30 cells). The viruses used were a SIV variant with a truncated TM glycoprotein and increased virion envelope glycoprotein content, SIVmac239/251 tail (lot P3973, produced from SUPT1/CCR5 CL.30 cells), a wild-type SIV with a full-length TM glycoprotein and lower envelope glycoprotein content, SIVmac2339 (lot P4118, produced from CEM X174(T1) cells), or wild-type HIV-1_MN_ (lot P4091, produced from SUPT1 cells). The virus SIVmac239/251 tail and the SUPT1/CCR5 CL.30 cells were provided by J. Hoxie (University of Pennsylvania). Cells were incubated with virus for 60 min at 4 °C, then warmed to 37 °C and maintained at this temperature until the conclusion of the experiment. After specific incubation periods, samples were fixed by addition of glutaraldehyde, to a final concentration of 2.5% by volume, in 0.1 M cacodylate buffer (pH 7.4) (Electron Microscopy Sciences, http://www.emsdiasum.com/microscopy/default.aspx). Cells incubated with virus in the presence of non-antibody inhibitors were processed in exactly the same way, except for the presence of either the TM peptide–derived fusion inhibitor SIVmac251 C34 (5 uM), or the CCR5 antagonist TAK779 (at 10 μM). For chronic infection samples, similar procedures were used for initial virus incubation, with an innoculum of SIVmac239/251 tail (lot P3973) corresponding to ∼600 ng p28^CA^ equivalent and 6 × 10^9^ virions, and a nominal multiplicity of infection of ∼0.05. Inoculated cells were washed twice, resuspended at 1 × 10^6^ cells/mL, and cultured for 3 d, at which time fresh medium was added and the cells incubated for an additional 4 d. After aspiration of the bulk of the supernatant, the cell suspension was fixed with glutaraldehyde as in the case of acutely infected cells.

Fixed cells were treated with reduced osmium (1:1 mixture of 2% aqueous potassium ferrocyanide) as described previously [34], embedded in 2% agar, dehydrated in ethanol, and embedded in Epon resin. Then, 100-nm- to 150-nm-thick sections were collected on copper grids and stained with lead citrate. Electron tomography was carried out at room temperature using a Tecnai 12 electron microscope (FEI Company) operated at 120 kV. Methods for data collection and reconstruction were the same as those used for cryogenic specimens described above.

### Statistical analysis of spikes on SIV tomograms.

To study the patterns of viral spike distribution, viral membranes were first segmented semi-automatically using energy-based segmentation algorithms that allow incorporation of shape and smoothness constraints to facilitate segmentation. Individual spikes were located automatically on the surface of the viral membrane, and their locations stored for subsequent computational analysis. Localization of viral membranes is most reliable in the central portion of viruses where the membranes are aligned to the best spatially resolved plane in the tomograms; we therefore used only a band comprising about one third of the total viral surface area for quantitative analysis of spike distribution.

Individual spikes were located automatically by template matching using a spherical template with a diameter of ∼10 nm. The search was restricted to identify features originating on the membrane surface and radiating outward. This procedure assigns a template-fitness value to all points on the membrane; this parameter is locally maximized at the location of viral spikes (represented with arrows pointing in the normal direction, see Figure 2). Once all spikes are located, distances between pairs of positions were measured along the surface of the viral membrane. To analyze the distribution of distances, we computed for all spikes the distance at which the three closest spikes were located, and plotted this distribution as a histogram.

## Abbreviations

3-D: three-dimensional
SIV: simian immunodeficiency virus
SU: surface glycoprotein
TM: transmembrane glycoprotein
